# Risk bases can complement dose bases for implementing and optimising a radiological protection strategy in urgent and transition emergency phases

**DOI:** 10.1007/s00411-019-00809-x

**Published:** 2019-07-25

**Authors:** Linda Walsh, Alexander Ulanowski, Jan Christian Kaiser, Clemens Woda, Wolfgang Raskob

**Affiliations:** 1 Department of Physics, Science Faculty, University of Zürich, Winterthurerstrasse 190, 8057 Zürich, Switzerland; 2 Institute of Radiation Medicine, Helmholtz Zentrum München – German Research Center for Environmental Health, Ingolstädter Landstraße 1, 85764 Neuherberg, Germany; 3 IAEA Laboratories, International Atomic Energy Agency, 2444 Seibersdorf, Austria; 4 Institute for Nuclear and Energy Technologies, Karlsruhe Institute of Technology, Hermann-von-Helmholtz Platz 1, 76344 Eggenstein-Leopoldshafen, Germany

**Keywords:** Nuclear accidents, Health risk assessment, Radiological emergency response, Radiation protection, Lifetime risk

## Abstract

Current radiological emergency response recommendations have been provided by the International Commission on Radiological Protection and adopted by the International Atomic Energy Agency in comprehensive Safety Standards. These standards provide dose-based guidance for decision making (e.g., on sheltering or relocation) via generic criteria in terms of effective dose in the range from 20 mSv per year, during transition from emergency to existing exposure situation, to 100 mSv, acute or annual, in the urgent phase of a nuclear accident. The purpose of this paper was to examine how such dose reference levels directly translate into radiation-related risks of the main stochastic detrimental health effects (cancer). Methodologies, provided by the World Health Organization after the Fukushima accident, for calculating the lifetime and 20 year cancer risks and for attributing relevant organ doses from effective doses, have been applied here for this purpose with new software, designed to be available for use immediately after a nuclear accident. A new feature in this software is a comprehensive accounting for uncertainty via simulation technique, so that the risks may now be presented with realistic confidence intervals. The types of cancer risks considered here are time-integrated over lifetime and the first 20 years after exposure for all solid cancers and either the most radiation-sensitive types of cancer, i.e., leukaemia and female breast cancer, or the most radiation-relevant type of cancer occurring early in life, i.e., thyroid. It is demonstrated here how reference dose levels translate differently into specific cancer risk levels (with varying confidence interval sizes), depending on age at exposure, gender, time-frame at-risk and type of cancer considered. This demonstration applies German population data and considers external exposures. Further work is required to comprehensively extend this methodology to internal exposures that are likely to be important in the early stages of a nuclear accident. A discussion is provided here on the potential for such risk-based information to be used by decision makers, in the urgent and transition phases of nuclear emergencies, to identify protective measures (e.g., sheltering, evacuation) in a differential way (i.e., for particularly susceptible sub-groups of a population).

## Introduction

The existing system of radiation protection, based on the International Commission on Radiological Protection (ICRP) 2007 Recommendations (ICRP 2007) and specified in the International Atomic Energy Agency (IAEA) system of safety standards, requirements and guides (IAEA 2006, 2014, 2015), addresses requirements for protection in planned or existing exposure situations or in cases of nuclear or radiological incidents and emergencies. Protection actions following the emergencies can be attributed to two phases: an emergency or urgent phase during or immediately following the incident, which is characterised by a quickly varying situations and radiological dangers to be addressed, and a longer phase of transition to an existing exposure situation when the situation is under control. During the urgent and transition phases of a nuclear emergency, important decisions on implementing measures aimed at protecting affected populations, such as sheltering or relocation, need to be made quickly, effectively and incisively. The IAEA Safety Standards, including principles, requirements and guidelines, provide a uniform, internationally accepted, framework to implement protective and mitigating actions. The IAEA generic criteria for planning emergency response actions are typically formulated using constraints and limits defined using dosimetric bases as ranges of effective dose (IAEA 2015, 2016). The IAEA requirements, based on publications from the ICRP (2007), suggest a typical band of effective dose from 20 to 100 mSv to be used for planning in the emergency situation. The IAEA requirements provide a common framework for the Member States to develop their own national legislations on radiation protection, including norms and limits.

Constraints and limits are not necessarily to be defined in terms of dose units. Alternatively, they can be formulated as risk constraints, i.e., risks associated with radiological dangers (see, e.g., IAEA 2016). The aim of this paper was to demonstrate that, in taking decisions aimed at protecting affected populations from the main stochastic effects (cancer), risk bases can complement dosimetric bases in the decision making process.

Unfortunately, in the past, cancer risk assessment software was not designed to be available for use immediately after a nuclear accident. After the Fukushima nuclear accident on 11th March 2011 (Wakeford 2011), for example, there was a time interval of just under 2 years between accident occurrence and the publication of the World Health Organization (WHO) health risk assessment (WHO 2013) report. This long-time interval was due to the work-load, after the event, in assessing doses, developing a risk assessment framework and developing the risk assessment software (and that was without a full explicit mathematical treatment of risk uncertainties). In order to close such potential future time gaps between accidents and health risk assessments, by implementing lessons learned after Fukushima (Walsh 2016), the European Union-CONFIDENCE (Coping with uncertainty for improved modelling and decision making in nuclear emergencies) project provided funding to develop a risk assessment software (the EU-CONFIDENCE software tool) designed to be immediately available after a nuclear accident. The tool encompasses the risk assessment methodological framework for assessing cancer risks after the Fukushima accident as suggested by a WHO expert group (WHO 2013; Walsh et al. 2014) and by the German software tool ProZES (Jacob et al., 2017; Ulanowski et al. 2016).

The WHO methodology has been applied to: (a) translate the effective dose limits to the organ doses that are relevant for radiation risk assessment; and (b) to calculate risk of all solid, breast, thyroid cancers and leukaemia using these converted organ dose and contemporary models of radiation risk for an illustrative modern European population; namely all calculations have been performed using the population data and disease statistics for Germany.

One new feature of the tool is a full mathematical treatment of uncertainties in the calculated risks, so that the risks can now be given with confidence intervals. Although German population data are considered here for illustration of the dose to risk conversions, the software tool also incorporates data for four Nordic countries and Switzerland and can be routinely extended with data for other countries.

It is this new software tool, which has been applied for the calculations presented here to show how the dosimetric limits can translate into cancer risk estimates and risk uncertainty. It is shown here that any one particular reference dose limit will translate differently into risks from stochastic effects depending on age at exposure, gender, the “at-risk” time-frame considered and cancer risk type. The potential of risk assessment tools that have been fully developed and ready for operation, before any nuclear accident actually takes place, is discussed here along with the idea to incorporate such a tool into currently available dosimetric large-area monitoring systems, e.g., the Java-based real-time on-line decision support system (JRODOS) (Ehrhardt and Weis 2000; Ievdin et al. 2010). The JRODOS system has been developed for general application worldwide for use in national or regional nuclear emergency centres. JRODOS provides coherent support at all stages of an accident (i.e., before, during and after a radiological release), including the long-term management and restoration of contaminated areas. The system is able to support decisions about the introduction of a wide range of potentially useful countermeasures (e.g., sheltering and evacuation of people, distribution of iodine tablets, food restrictions, agricultural countermeasures, relocation, decontamination, restoration, etc.) mitigating the consequences of an accident with respect to detrimental health effects, the environment, and the economy. JRODOS can be applied to accidental releases into the atmosphere and into various aquatic environments. Appropriate interfaces exist with local and national radiological monitoring data, meteorological measurements and forecasts, and for adaptation to local, regional and national conditions.

Detailed discussions are provided on how such risk information, including the relevant uncertainty of this risk information, could potentially be useful for integrating into the radiation protective decision making processes after a nuclear accident.

## Materials and methods

Radiation-related cancer risks were estimated for both males and females initially exposed as infants (age 1 year), children (age 10 years) or adults (age 20 years). Models for specific cancer sites were applied to calculate risks attributable to radiation over a lifetime and over the initial 20 years after the nuclear accident, based on generic recommended reference limits of effective dose converted to organ/tissue dose, and using demographic and health statistics data from a contemporary illustrative European population (German population).

### Effective dose conversion to organ/tissue dose

Current IAEA safety requirements in their parts 3 and 7 (IAEA 2014, 2015) give generic criteria for use in conjunction with the goals of emergency response in terms of effective dose in the range 20–100 mSv, acute or annual, that includes dose contributions via all exposure pathways.

The ICRP has expressed caution in the use of effective dose for purposes of estimating risks to individuals or populations exposed to ionising radiation, especially for very heterogeneous exposures in medical procedures and environmental or occupational exposure to, for example, radioisotopes of iodine (International Commission on Radiological Protection 2007, paragraph 151). Therefore, in order to calculate the risks corresponding to these IAEA reference levels of 100 mSv, acute or annual, in the urgent phase and an effective dose of 20 mSv per year in the transition phase it is necessary to convert these levels into organ/tissue doses for each of the target organs for the types of cancers evaluated (i.e., colon, red bone marrow, thyroid and breast organ/tissue doses for all solid cancer, leukaemia, thyroid and breast cancer, respectively).

A methodology that can be applied to calculate organ doses from effective doses for the general population has already been presented (WHO 2013, Annex G, p. 133). In this WHO methodology the organ dose coefficients have been deduced using relationships between effective and organ doses for age-dependent gender-specific human models (phantoms) exposed to external sources of low-LET radiation (photons) presented by Jacob et al. (1990), Petoussi-Henss et al. (2012) and Saito et al. (2012). In an emergency situation, the organ doses could result from four possible pathways: (a) external exposure to radioactive materials deposited on the ground; (b) external exposure to the release plume or radioactively contaminated ambient air; (c) internal exposure due to inhalation of radioactively contaminated air; and (d) internal exposure due to ingestion of radionuclides. The relative contributions to the total organ doses via each of these four pathways will be highly variable and dependent on the nature of the accident, peculiarities of radioactive contamination of the environment and human habitats, protective actions taken or other factors; therefore, no generic solution can be found without consideration of the specific exposure scenario.

In order to illustrate the principle of general consideration of risk bases for decision making, a simplifying assumption is made here that either an acute or annual (first-year) effective dose just comes from external exposures to gamma radiation under a scenario equivalent to that realised after the Fukushima accident (the limitations of such an important assumption are fully given in the discussion section). Under this assumption, only external exposure to low-LET radiation (photons and electrons with radiation weighting factor equal to one) is relevant and the organ absorbed doses (Gy) are numerically equal to the organ equivalent doses (Sv). The latter have been deduced from the effective dose in the WHO 2013 Report (see WHO 2013, Annex G, Table 19, p. 134) and are used in this paper.

For the radionuclide composition specific to the Fukushima accident, the deduced ratios of the organ and effective dose are very close to one, spanning the range from 0.89 (adult, red bone marrow) to 1.0, so uncertainty associated with conversion of the effective dose coefficients is generally low, where external gamma radiation exposures are concerned, under this scenario. The UNSCEAR 2013 Report has applied a similar approach using data from the ICRP Publication 74 (ICRP 1996) and the more recent results of Petoussi-Henss et al. (2012) and arrived at similar results: all differences between age-dependent organ absorbed and effective doses did not exceed 5–10% (UNSCEAR 2014, Attachment C-12).

### Health statistics data

Population cancer incidence and mortality rates, given by sex, cancer site and 5-year age group, for 2014, are available from the German cancer register (RKI-GEKID 2017). All-cause mortality rates for 2013/2015 and general survival data from life tables for Germany are available from the German Federal Office for Statistics (Statistisches Bundesamt 2016). Similar data are already included for Denmark, Finland, Norway, Sweden and Switzerland.

### Risk models for specific cancer sites

The following malignant diseases or groups of malignant diseases were considered (ICD-10 classification codes are shown in parentheses):

- All solid cancers (C00–C80);
- Leukaemia, defined here as all leukaemia (i.e., most of the ICD10:C91–C95 subclasses, excluding CLL, C91.1 and C91.4, and excluding ATL, C91.5);
- Female breast cancer (C50);
- Thyroid cancer (C73).

These groupings have been demonstrated to show a radiation risk effect modification by age-at-exposure (UNSCEAR 2013). The grouping “all solid cancer” (ICD10:C00–C80) was included to address the overall cancer risk from radiation, because radiation can cause cancer in most organs/tissues of the body, and to provide risk estimates based on a large outcome grouping with a higher statistical power than otherwise obtainable just from analyses on individual cancer sites. The group of leukaemia, including all types of leukaemia without CLL and without ATL, referred to hereafter as “leukaemia”, was considered due to their known radiation sensitivity and short latency period, thus potentially becoming one of the first effects to be observed following radiation exposure. Significant increases in the number of thyroid cancers following exposure in childhood was shown as a major radiological consequence of the Chernobyl accident, so thyroid cancer is also considered here (UNSCEAR 2008, Vol. II, Annex D). Breast cancer is the most common female cancer worldwide and is a leading cancer mortality cause among women. Breast tissue is also considered to be particularly radiosensitive at young ages at exposure, so the effect of radiation exposure on this type of cancer should also be considered.

The models of radiation risk, establishing relationships between the risk for the cancer type groupings given above and dose from exposure, were taken from publications related to the Japanese A-bomb survivor Life Span Study (LSS) cohort (the basis for this selection is given in the discussion section). These risk to dose response models are in terms of excess relative risk (ERR) and/or excess absolute risk (EAR) with the following: a follow-up 1958–2001 taken from Table 3 of Hsu et al. (2013) (i.e., models for the grouping leukaemia minus CLL and minus ATL, based on 312 cases); a follow-up 1958–1999 taken from Tables S2 and S3 of Jacob et al. (2014), for thyroid cancer; a follow-up 1958–2009 taken from Table 5 of Grant et al. (2017), for all solid cancers unadjusted for smoking (although Grant et al. 2017 did not publish an EAR model unadjusted for smoking that would have been suitable for this application—the authors have provided such a model in “Appendix”). The model for breast cancer was taken from a pooled study of eight cohorts, i.e., the model in Table 12 of Preston et al. (2002), with the full parameter set given in Jacob et al. (2017), where the LSS cohort contributes almost 60% of cases. All these models have similar properties: a linear dose–response function for all solid cancers, thyroid cancer and female breast cancer and a linear-quadratic dose–response function for leukaemia; including risk effect modification by age-at-exposure (*e*), sex (*s*) and attained age (*a*).

To at least partially account for uncertainty associated with model selection, the Multi-Model Inference (MMI) technique (see, e.g., Burnham and Anderson 2002) was used wherever possible. Namely, for each of the two groupings all solid cancers (Grant et al. 2017) and leukaemia cancers (Hsu et al. 2013), two models, one of EAR-type and one of ERR-type, were used for risk calculations with relative weights based on the Akaike Information Criterion (AIC) (as in Walsh and Schneider 2013). The EAR-type model of Preston et al. (2002) for breast cancer was developed from a pooled cohort and no updated alternative models were found for this pooled cohort at the time of writing. The thyroid cancer risk model of Jacob et al. (2014) was of ERR-type. The excess and baseline incidence rates provided by the risk models are pertinent to their respective epidemiological cohort—for the considered models, this is mainly the LSS cohort. To estimate radiation risks for the target population, i.e., population of interest, these rates need to be “transferred”, which means that the estimated radiation-attributed excess rate is transformed using the ratio of the model and the population baseline rates assuming either additive (the same EAR) or multiplicative (the same ERR) mechanisms of transfer. More on this procedure can be found in Ulanowski et al. (2016).

Following risk transfer, the resulting excess incidence rate, ${\text{ER}}\left( {d,e,a,s} \right)$, is given by

1 $${\text{ER}}\left( {d,e,a,s} \right) = f \,{\text{EAR}}\left( {d,e,a,s} \right) + \left( {1 - f} \right){\text{ERR}}\left( {d,e,a,s} \right)m\left( {a,s} \right),$$

where *f* is the weighting factor between an additive (EAR) and a multiplicative (ERR) transfer of risk; *m*(*a*, *s*) is the age- and sex-specific baseline cancer incidence rate in the target population. To allow for modelling uncertainty associated with unknown type of risk transfer mechanism the *f* values were part of the Monte Carlo simulation procedure with the sampling distribution assumed to be uniformly distributed in the range from 0 to 1.

For breast cancer, where only an EAR-type model was applied, as strongly recommended by Preston et al. (2002), the risk transfer was modelled assuming an unknown ratio of baselines in epidemiological cohorts and the target populations (see details in Ulanowski et al. 2016). For thyroid cancer, the risk transfer was modelled based on only an ERR model, as described in Jacob et al. (2014).

Computations in this paper have been performed using contemporary cancer incidence rates for an illustrative European population (Germany) in 2010–2014.

### Risk quantities

The conventional lifetime attributable risk (LAR) (Thomas et al. 1992; Vaeth and Pierce 1990) was selected as the risk quantity for application here. LAR closely approximates the risk of exposure-induced death from (REID) or incidence of (REIC) cancer, and other similar measures (Kellerer et al. 2001), at the doses relevant to protecting populations from stochastic effects following exposures with organ doses under about 0.5 Gy.

The central estimate for the attributable risk from either one annual dose or one acute dose, ${\text{AR}}\left( {d,e,s,a} \right)$, specifies the sex (*s*) and age-at-exposure (*e*) specific cumulative probability of a specific cancer attributable to radiation exposure with dose *d*. The AR involves integrating over time, *t*, from *e* up to an age *a*:

2 $${\text{AR}}\left( {d,e,s,a} \right) = \mathop \int \limits_{e}^{a} {\text{ER}}\left( {d,e,t,s} \right)\frac{{S_{aj} \left( {t,s} \right)}}{{S_{aj} \left( {e,s} \right)}}F_{\text{L}} \left( {t - e} \right){\text{d}}t,$$

Here *d* is the dose delivered to the organ/tissue at age-at-exposure *e*, and $F_{\text{L}} \left( {a - e} \right)$ is a function smoothly varying from 0 to 1 which models the effects of the unknown minimum latent times between the delivery of the dose to the organ and the expression of the radiation-related cancer risk.

If the integration of the risk is performed over the whole lifetime, then Eq. (2) converges to a conventional definition of LAR:

3 $${\text{LAR}}\left( {d,e,s} \right) = {\text{AR}}\left( {d,e,s,\infty } \right).$$

The latency function, $F_{\text{L}}$, for all solid cancers, including breast and thyroid cancers, grow from zero to one in the range, approximately, from 1.5 to 7 years since exposure, while reaching the value 0.5 at time 3.5 years since exposure (where these values were chosen with a consideration of recommended values (UNSCEAR 2008, BEIR VII—Phase 2 2006, Heidenreich 1999). For leukaemia, the minimum latency period is shorter, and the corresponding latency function grows from 0 to 1 in the range from 1 to 3 years since exposure, while having the value of 0.5 at time 1.5 years after exposure (again values were chosen to be consistent with recommended values (UNSCEAR 2013, Annex B)).

The conditional survival curve $S_{aj} \left( {t|e,s} \right) = S_{aj} \left( {t,s} \right)/S_{aj} \left( {e,s} \right)$, is the probability of surviving cancer-free to age *t* conditional on the probability to be alive and disease-free at the age of exposure *e*. $S_{aj} (t|e,s)$ was calculated from the German life tables as well as cause-specific incidence and mortality rates in 2014, as described above. LAR or AR was also compared with the lifetime or age-specific baseline risk (LBR or BR, correspondingly) in order to put radiation-related cancer risks into the perspective of the baseline cancer risk in Germany (i.e., the risk in the absence of radiation exposure from an accident). Applying the same notation as for definition of AR (Eq. 2), the BR and LBR conditional on disease-free survival to age *e* are calculated as follows:

4 $${\text{BR}}\left( {e,s,a} \right) = \mathop \int \limits_{e}^{a} m\left( {t,s} \right)S_{aj} \left( {t|e,s} \right){\text{d}}t\quad {\text{and}}\quad {\text{LBR}}\left( {e,s} \right) = {\text{BR}}\left( {e,s,\infty } \right).$$

The duration of any lifetime segment at-risk considered depends on the age at exposure (i.e., the higher the ages at initial exposure the shorter the lifetime segment up to old age). This causes any comparisons of results among different ages at exposure to be complicated. Therefore, the cumulative risks over 20 years-at-risk after the initial exposure (AR_20_, BR_20_) were also calculated. AR_20_ can be a suitable representation to satisfy interest in early risks of cancer from a short-term public health perspective and also for comparisons between calculated risks and risks potentially provided by any epidemiological studies initiated after an emergency. AR_20_ is particularly relevant for cancer types such as leukaemia and thyroid cancer where the relative increase in risk is expected to be stronger during the first few decades after exposure during childhood. It is pertinent to note that these risk quantities, although they can be based on individual doses, cannot represent an individual’s risk due in-part to a lack of knowledge on other individual risk factors (see discussion). The forms given in the Eqs. (2)–(4), which result in probability values for the various risk measures, are, therefore, more appropriate to consider in the results section, when simply converted into number of cases per 10,000 persons.

### Treatment of uncertainties in the risk calculations

Such lifetime risk estimates are associated with large uncertainties that were quantified here with stochastic simulation following a methodology that has recently been described for non-time-integrated risks (Ulanowski et al. 2016) and time-integrated thyroid cancer risks (Jacob et al. 2014, with full details in the supplementary material for this cited reference). The following uncertainties were included here in the simulation of overall risk uncertainties:

1. The radiation risk model parameters from the A-bomb LSS cohort and the pooled breast cancer cohorts were sampled from a multivariable normal distribution using best estimates of all the model parameters, including parameters specifying baseline incidence in the LSS cohort, and the respective full covariance matrices.
2. The transfer factors *f* (i.e., from Eq. 1, for apportioning additive and multiplicative radiation risk contributions) were sampled from a uniform distribution: $f \sim U\left( {0,1} \right)$, thus corresponding to highest uncertainty of risk transfer.
3. Dose rate effects were sampled from a lognormal distribution with a geometric mean of 1.0 and geometric standard deviation varying as a linear function of dose rate (Jacob et al. 2014, 2017) with value of 1.5 at dose rate 1.5 mGy day^−1^ and value of 1 at dose rate equal to or higher than 6 mGy h^−1^. Correspondingly, the median dose rate correction factor does not change but results in a higher variance at lower dose rates.
4. The minimum latency periods were sampled from a sigmoid distribution with parameters suggested by I. Apostoaei (ORRISK, USA) and found in Jacob et al. (2017).
5. Uncertainty of incidence data was sampled from Poisson distributions of the reported number of cancer cases in a country in the corresponding 5-year age interval.
6. The doses were sampled from a log-normal distribution, with arithmetic means of 20 and 100 mSv (converted into organ doses appropriate to the cancer outcome type considered, Table 1). The geometric standard deviations were assumed here to be 1.5 (see, e.g., Harada et al. 2014) and the arithmetic mean organ dose values were converted to geometric means to account for the known inequality between these two quantities.

**Table 1:** The organ dose ranges corresponding to an effective dose range of 20–100 mSv, calculated with the ratio of organ to effective dose, from external exposures, for the situation after the Fukushima accident, as given in Table 19 of the WHO report (2013, Annex G, p. 134)

Age at exposure (year)	Organ dose ranges (mSv) corresponding to an effective dose range of 20–100 mSv			
	Breast	Colon	RBM	Thyroid
20 (adult)	19.8–99	18.2–91	17.8–89	20–100
10 (child)	20–100	19.2–96	20–100	20–100
1 (infant)	20–100	18.2–91	18.8–94	20–100

## Results

Table 1 gives the organ dose ranges corresponding to an effective dose range of 20–100 mSv, calculated with the ratios of organ to effective dose from external exposures, taken from Table 19 of the WHO report (2013, Annex G, p. 134).

**Fig. 1:**
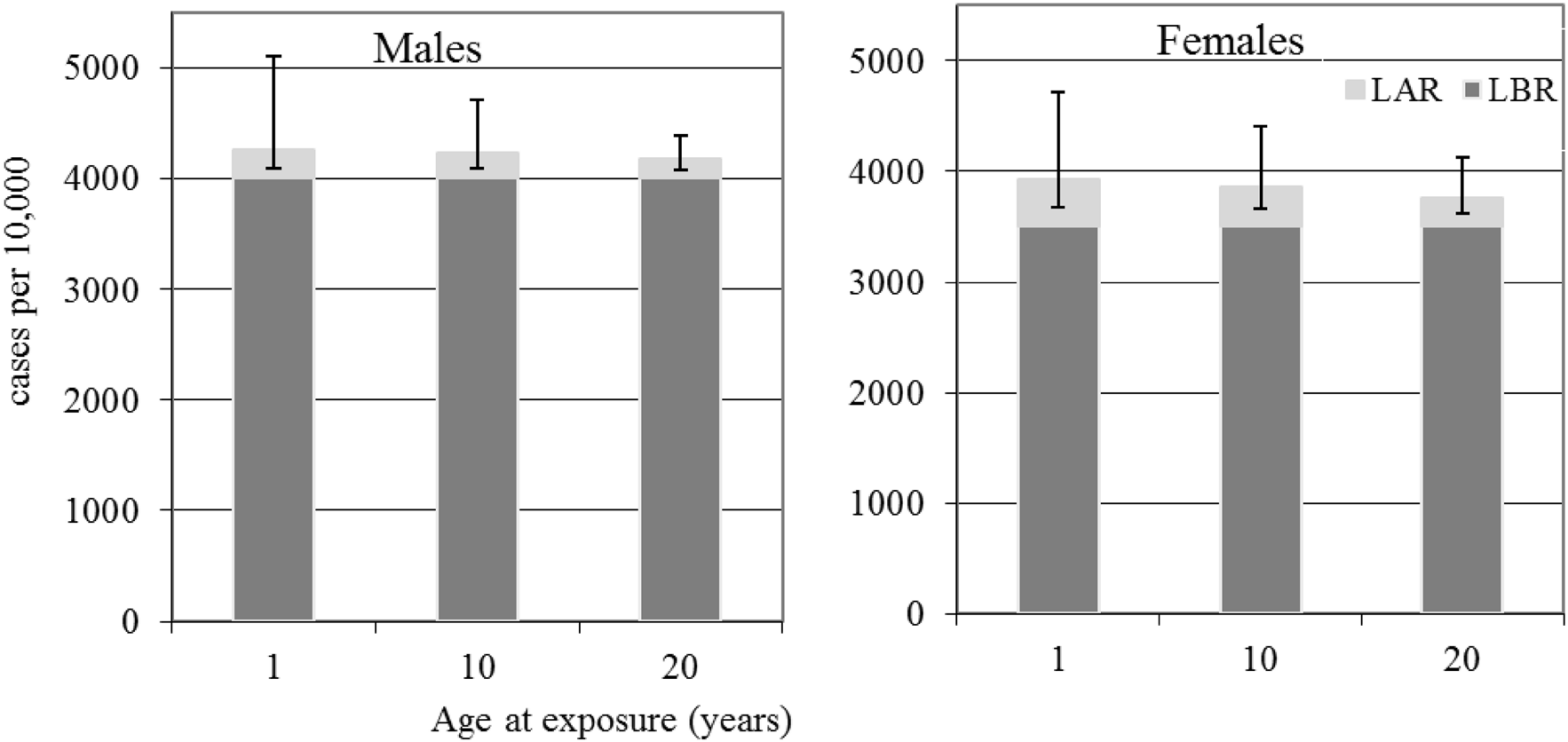
Male and female all solid cancer baseline (dark grey) and radiation (light grey with error bars) risks in cases per 10,000 persons calculated from LBR and the LAR for 100 mSv effective dose. Error bars are for 95% confidence intervals

**Fig. 2:**
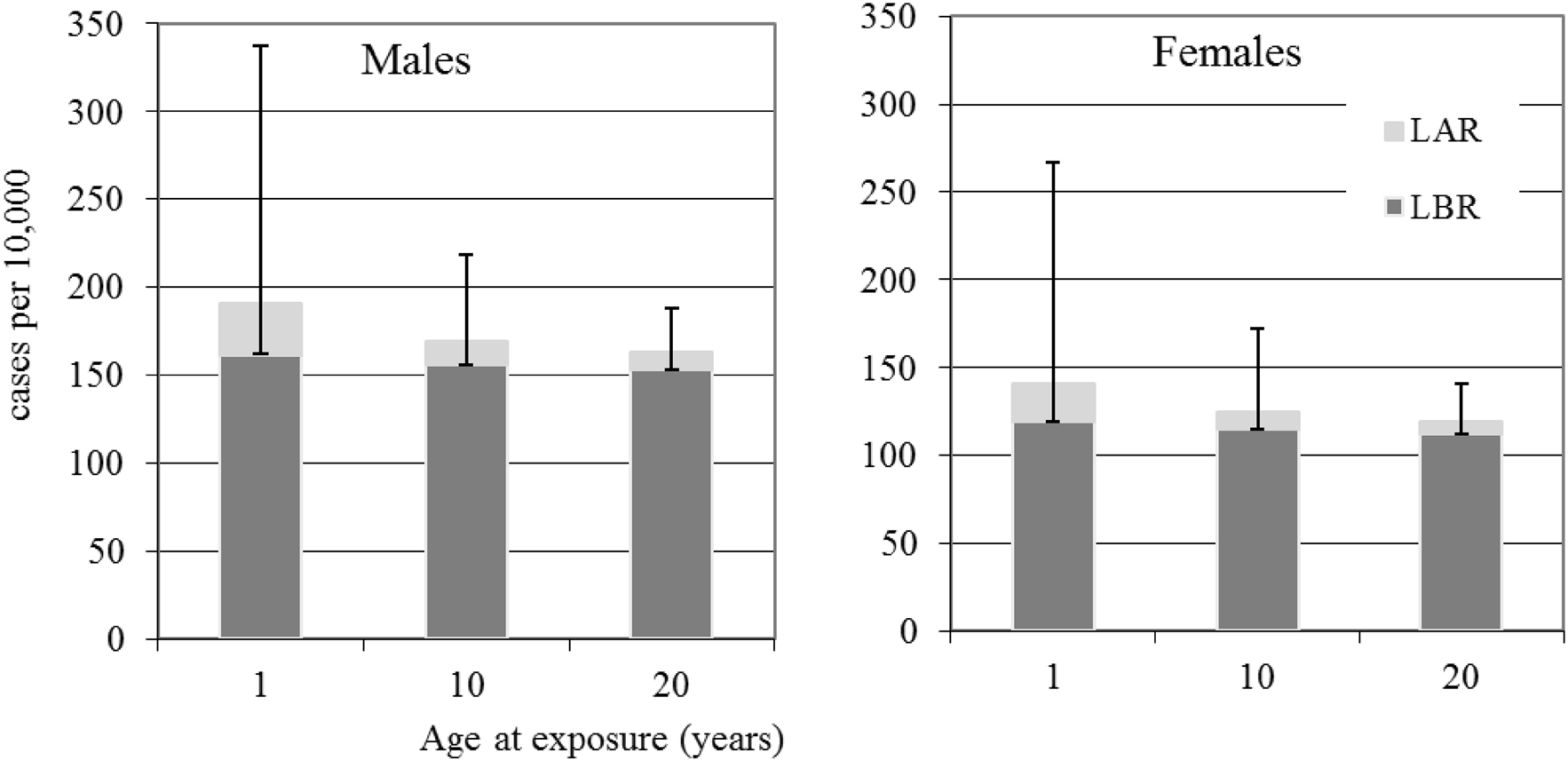
Male and female leukaemia baseline (dark grey) and radiation (light grey with error bars) risks in cases per 10,000 persons calculated from LBR and the LAR for 100 mSv effective dose. Error bars are for 95% confidence intervals

**Fig. 3:**
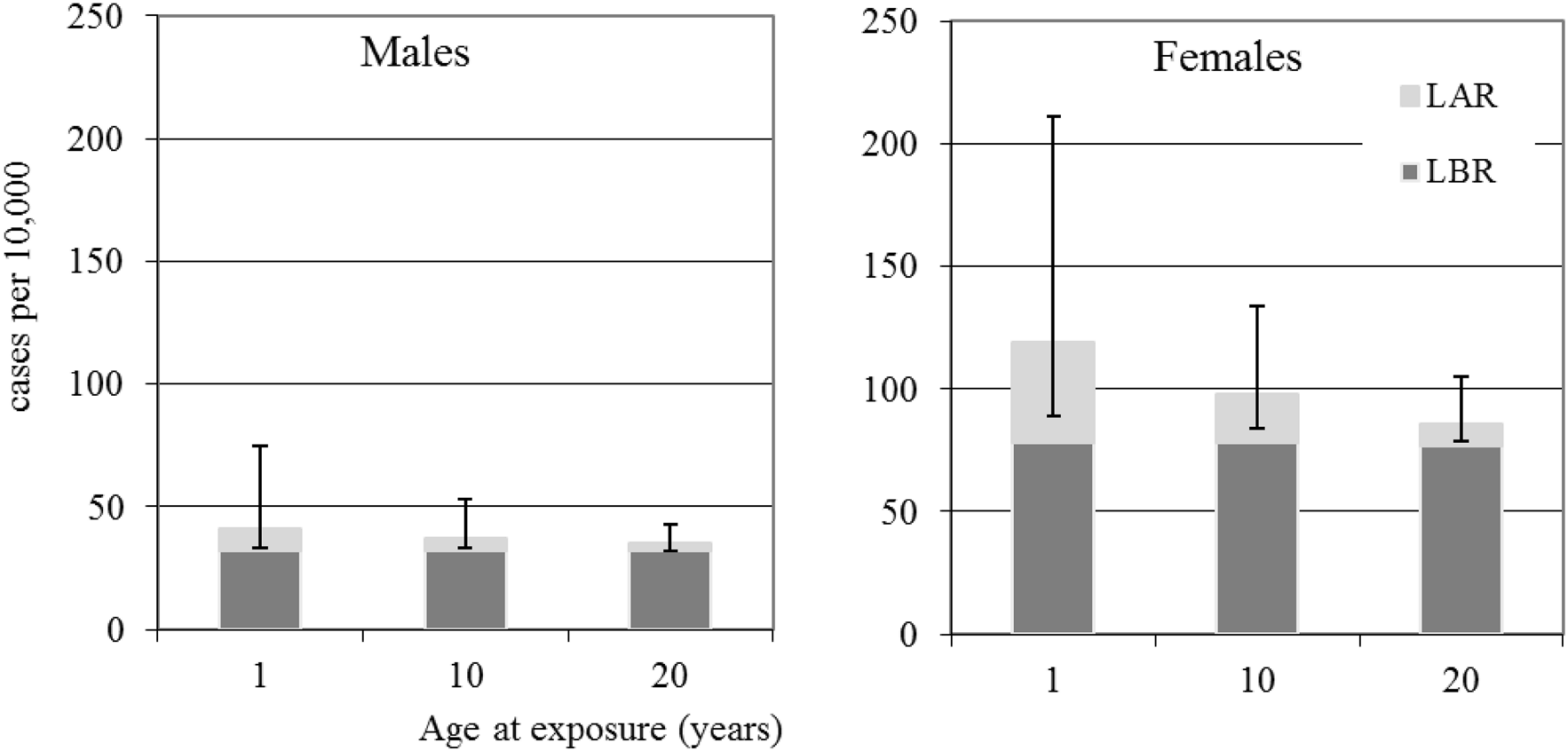
Male and female thyroid cancer baseline (dark grey) and radiation (light grey with error bars) risks in cases per 10,000 persons calculated from LBR and the LAR for 100 mSv effective dose. Error bars are for 95% confidence intervals

**Fig. 4:**
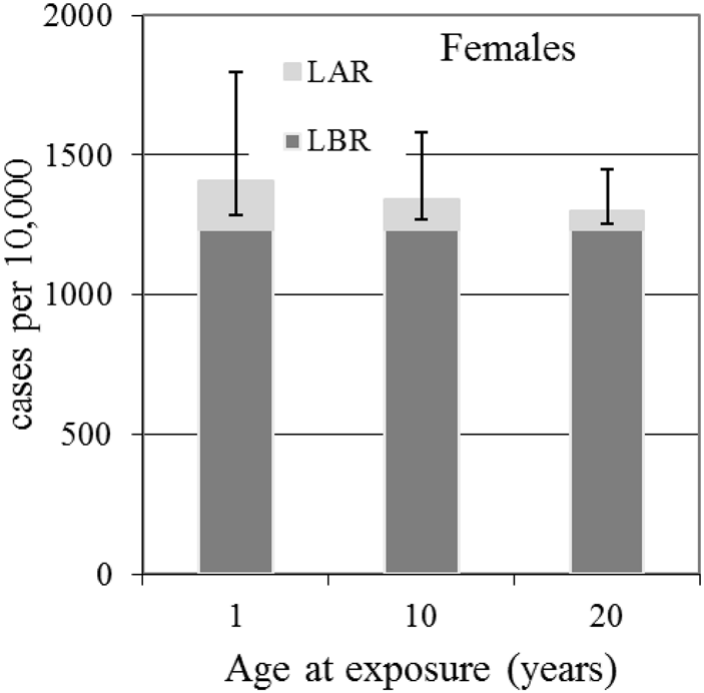
Female breast cancer baseline (dark grey) and radiation (light grey with error bars) risks in cases per 10,000 persons calculated from LBR and the LAR for 100 mSv effective dose. Error bars are for 95% confidence intervals

The LAR, LBR, AR_20_ and BR_20_ risks (i.e., integrated over lifetime and the first 20 years at-risk since exposure) which have been simply converted from probabilities to numbers of excess cases and number of baseline cases per 10,000 persons, for age at exposure 1, 10 and 20 years, are given in Tables 2, 3, 4 and 5, and Figs. 1, 2, 3 and 4, for all solid cancers, leukaemia, thyroid and female-breast cancers, respectively.

**Table 2:** All solid cancer, ranges for median number of cases per 10,000 persons after 20 years and during lifetime based on German population data for 20–100 mSv effective dose range

		Ranges for median numbers of cases per 10,000 (with 95% CI) after 20 years-at-risk since exposure and during lifetime simply converted (10,000 times risk) from the risks in column 2			
Age at exposure (years)	Sex	Male		Female	
	Effective dose	20 mSv	100 mSv	20 mSv	100 mSv
20 (adult)	AR_20_	2 (1; 14)	11 (3; 70)	5 (2; 13)	25 (10; 63)
	LAR	33 (15; 76)	166 (73; 381)	51 (22; 123)	257 (112; 618)
	BR_20_	102 (90; 116)		164 (149; 181)	
	LBR	4005 (3878; 4140)		3509 (3385; 3639)	
10 (child)	AR_20_	2 (0; 40)	10 (2; 200)	3 (1; 9)	17 (6; 43)
	LAR	46 (18; 143)	229 (92; 716)	70 (30; 179)	350 (149; 895)
	BR_20_	45 (37; 54)		50 (42; 60)	
	LBR	4002 (3873; 4140)		3509 (3383; 3643)	
1 (infant)	AR_20_	1 (0; 29)	5 (0; 144)	2 (1; 8)	11 (3; 41)
	LAR	53 (18; 219)	264 (88; 1097)	83 (33; 240)	416 (164; 1200)
	BR_20_	20 (14; 27)		18 (13; 26)	
	LBR	4002 (3871; 4143)		3511 (3383; 3647)	

All of the tabulated results come from Monte-Carlo simulations and are, therefore, subject to statistical fluctuations

**Table 3:** Leukaemia, ranges for median number of cases per 10,000 persons after 20 years and during lifetime based on German population data for 20–100 mSv effective dose range

		Ranges for median numbers of cases per 10,000 (with 95% CI) after 20 years-at-risk since exposure and during lifetime simply converted (10,000 times risk) from the risks in column 2			
Age at exposure (years)	Sex	Male		Female	
	Effective dose	20 mSv	100 mSv	20 mSv	100 mSv
20 (adult)	AR_20_	1 (0; 3)	4 (0; 14)	0 (0; 2)	2 (0; 11)
	LAR	2 (0; 7)	10 (0; 35)	1 (0; 5)	7 (0; 29)
	BR_20_	7 (4; 11)		5 (3; 8)	
	LBR	153 (129; 184)		112 (92; 138)	
10 (child)	AR_20_	1 (0; 6)	6 (0; 33)	1 (0; 5)	4 (0; 29)
	LAR	2 (0; 11)	13 (0; 62)	2 (0; 10)	10 (0; 57)
	BR_20_	7 (4; 11)		4 (2; 8)	
	LBR	156 (131; 190)		115 (94; 143)	
1 (infant)	AR_20_	3 (0; 25)	19 (0; 138)	2 (0; 20)	14 (0; 112)
	LAR	6 (0; 33)	30 (1; 176)	4 (0; 28)	22 (0; 148)
	BR_20_	9 (6; 14)		7 (4; 12)	
	LBR	161 (134; 197)		119 (96; 149)	

All of the tabulated results come from Monte-Carlo simulations and are, therefore, subject to statistical fluctuations

**Table 4:** Thyroid cancer ranges for median number of cases per 10,000 persons after 20 years and during lifetime based on German population data for 20–100 mSv effective dose range

		Ranges for median numbers of cases per 10,000 (with 95% CI) after 20 years-at-risk since exposure and during lifetime simply converted (10,000 times risk) from the risks in column 2			
Age at exposure (years)	Sex	Male		Female	
	Effective dose	20 mSv	100 mSv	20 mSv	100 mSv
20 (adult)	AR_20_	0 (0; 1)	1 (0; 3)	0 (0; 2)	2 (0; 9)
	LAR	1 (0; 2)	3 (0; 11)	2 (0; 6)	9 (2; 28)
	BR_20_	5 (3; 9)		19 (14; 25)	
	LBR	32 (22; 48)		77 (61; 101)	
10 (child)	AR_20_	0 (0; 1)	1 (0; 4)	0 (0; 2)	2 (0; 10)
	LAR	1 (0; 4)	5 (1; 21)	4 (1; 11)	20 (6; 56)
	BR_20_	2 (1; 5)		8 (5; 12)	
	LBR	32 (22; 49)		78 (61; 103)	
1 (infant)	AR_20_	0 (0; 1)	1 (0; 5)	0 (0; 1)	2 (0; 7)
	LAR	2 (0; 9)	9 (1; 43)	8 (2; 27)	41 (11; 133)
	BR_20_	1 (0; 2)		2 (1; 4)	
	LBR	32 (22; 50)		78 (61; 104)	

All of the tabulated results come from Monte-Carlo simulations and are, therefore, subject to statistical fluctuations

**Table 5:** Breast cancer ranges for median number of cases per 10,000 persons after 20 years and during lifetime based on German population data for 20–100 mSv effective dose range

	Ranges for median numbers of cases per 10,000 (with 95% CI) after 20 years-at-risk since exposure and during lifetime simply converted (10,000 times risk) from the risks in column 2		
Age at exposure (years)	Sex	Female	
	Effective dose	20 mSv	100 mSv
20 (adult)	AR_20_	1 (0; 3)	4 (1; 14)
	LAR	13 (4; 42)	65 (20; 211)
	BR_20_	53 (45; 62)	
	LBR	1235 (1161; 1313)	
10 (child)	AR_20_	0 (0; 2)	2 (1; 8)
	LAR	22 (7; 69)	109 (34; 345)
	BR_20_	7 (5; 10)	
	LBR	1234 (1160; 1312)	
1 (infant)	AR_20_	–	–
	LAR	35 (10; 113)	173 (51; 566)
	BR_20_	0 (0; 1)	
	LBR	1232 (1159; 1310)	

All of the tabulated results come from Monte-Carlo simulations and are, therefore, subject to statistical fluctuations

Considering the risk of all solid cancers (Table 2 and Fig. 1), it can be seen that adult females have a larger radiation risk than adult males, over the first 20 years-at-risk since exposure and over lifetime, but male adults have a lower baseline risk over the first 20 years-at-risk since exposure, but a higher lifetime baseline risk than females. For children and infants, the lifetime and 20-year radiation risk is higher for females than for males. Given that the grouping “all solid cancer” will provide risk estimates with higher statistical power than obtainable with individual cancer sites it is noteworthy that the 95% confidence intervals on the numbers of cases expected per 10,000 persons at 100 mSv over lifetime are still large, e.g., 416 (95% CI 164; 1200), 350 (95% CI 149; 895) and 257 (95% CI 112; 618) for females exposed as infants, children and adults, respectively.

The trends apparent from Table 3 and Fig. 2 for leukaemia, in the radiation risk sex differences, reflect those differences reported in the ERR and EAR LSS risk models (Hsu et al. 2013), i.e., the ERR model did not support a gender effect but the EAR did, with a female to male ratio of 0.66. Due to the equal probability of additive and multiplicative transfer types in the LAR calculations applied here, it can be seen that the male radiation risks are consistently slightly higher than the female risks at the same doses and for all ages at exposure considered. The male leukaemia baseline risk over the first 20 years-at-risk since exposure and over lifetime are also consistently higher than the female risks for all three ages at exposure considered. It can also be seen from Table 3, by comparing the numbers of cases per 10,000 persons after 20 years-at-risk since exposure with the numbers of cases per 10,000 during lifetime, that a substantial proportion of the overall radiation risk is accumulated in the first 20 years-at-risk since exposure: for adults and children, just under half of the lifetime risk from 100 mSv is accumulated in the first 20 years-at-risk; and for infants about two-thirds of the lifetime risk from 100 mSv is accumulated in the first 20 years-at-risk.

Considering the risks for thyroid cancer given in Table 4 and Fig. 3, it can be seen that females have higher radiation risks at the same dose and higher baseline risks than males. The numbers of cases expected per 10,000 at 100 mSv over lifetime is 41, 20 and 9 for females exposed as infants, children and adults, respectively. The numbers of cases expected per 10,000 at 100 mSv over lifetime is 9, 5 and 3 for males exposed as infants, children and adults respectively. However, it can be seen from Table 4 and Fig. 3 that the uncertainties on these expected numbers of cases are large.

Table 5 and Fig. 4 show, for female breast cancer, that the numbers of cases expected per 10,000 for an exposure of 100 mSv over lifetime is 173, 109 and 65 for exposure as infants, children and adults, respectively.

**Fig. 5:**
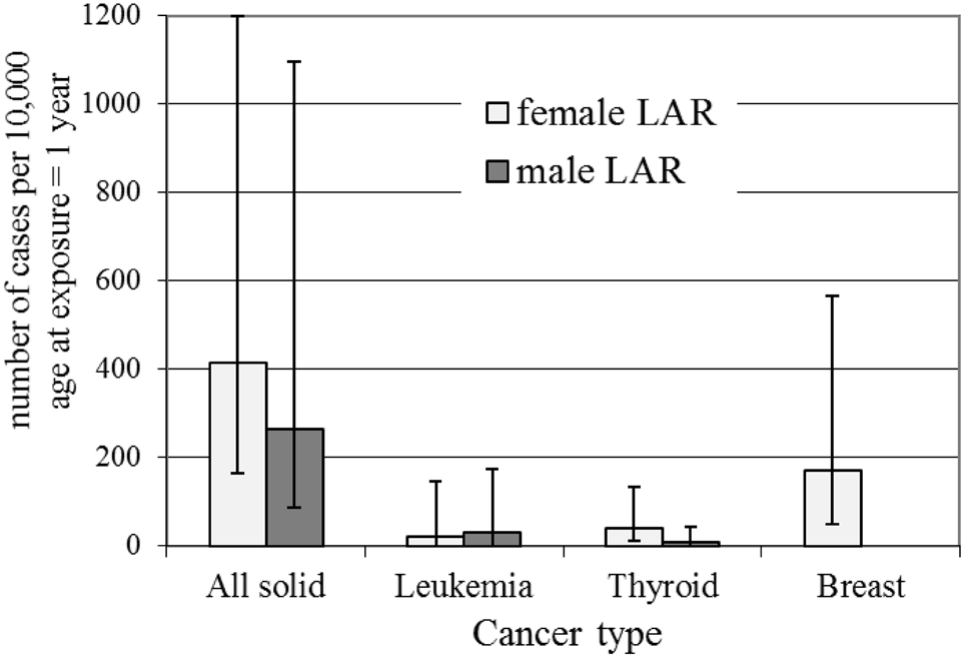
Cases per 10,000 persons for females (light grey) and males (dark grey) for different types of cancer, calculated from the radiation risks, LAR for 100 mSv effective dose for age at exposure of 1 year. Error bars are for 95% confidence intervals

Compared to the numbers of cases for female all solid cancers expected per 10,000 at 100 mSv over lifetime of 416, 350 and 257 for exposure as infants, children and adults, respectively—the breast cancer risk represents a substantial fraction of the total all solid cancer risk. This feature of the results can also be seen from Fig. 5 which shows the expected number of cases per 10,000 persons for different types of cancer, calculated from the LAR for 100 mSv effective dose for age at exposure of 1 year. From Fig. 5 it can also be seen that in absolute terms, the risks for thyroid cancer and leukaemia are much smaller than for all solid cancer and female breast cancer.

## Discussion

In the past, cancer risk assessment software was not designed to be fully developed and ready for operation, before a nuclear accident actually took place. Therefore, after the Fukushima nuclear accident in March 2011 there was no suitable software available for immediate use. The time intervals between the Fukushima nuclear accident on 11th March 2011 and the publication of various international health risk assessments, such as those from the WHO (2013) or UNSCEAR (2014), of just under 2 years and just over three and a half years, respectively, is illustrative of this situation. Such long time intervals are generally due to the work-load, after the event, in assembling expert groups, assessing doses, developing a risk assessment framework and developing the risk assessment software.

There is, therefore, a great potential for risk assessment tools that have been fully developed and are ready for operation, before any nuclear accident actually takes place. The probability for such an accident to happen in the next decades is not negligible (Kaiser 2012). Such potential is even greater, if the risk assessment tools can be either directly integrated into, or used in tandem with, currently available dosimetric large-area monitoring systems (e.g., JRODOS, Ehrhardt and Weis 2000; Ievdin et al. 2010).

JRODOS so far provides only dose-based results as input to the decision making process. Doses might be either based on prognostic calculations applying an estimated source term and numerical weather prognosis data or on available monitoring information. The monitoring information is point based but, within the EU-CONFIDENCE project, interpolation schemes are under development to provide aerial information from the prognostic calculations. However, as discussed above, risk-based assessments are important for estimating health effects and deciding on interventions, e.g., on medical screening actions. Effective medical screening has to be initiated early after the emergency and thus risk-based approaches, complementary to monitoring and prognosis results, will improve the potential in decision making.

In providing a framework and a software for use in health risk assessment, it is important to stress the necessity of avoiding any misunderstandings in the interpretation of the risks calculated. Risks in terms of lifetime attributable risks, LAR, calculated here, although they could be based on individual doses, cannot represent an individual’s cancer risks. This is because there is generally no information on important co-factors that influence a particular individual’s cancer risk such as the following: individual radiation sensitivity; any genetic pre-disposition to cancer development; lifestyle factors such as smoking status and alcohol intake; occupational risk factors; and past medical conditions treated with chemotherapy or radiation. Furthermore, population-based incidence and survival curves, used in the integration of risks over time, only represent average values for the national population considered. With all of these factors considered, LAR and AR_20_ should, therefore, be interpreted as an average risk for specific ages at exposure and genders. So, for example, an all solid cancer incidence LAR of 0.0264 (probability) for a male exposed to 100 mSv at age 1 year, should not be interpreted as an individual’s risk, but must be seen statistically—out of 10,000 males exposed at age 1 year to 100 mSv effective dose, there is a probability that, on average, 264 (note the wide 95% confidence intervals ranging from 88 to 1097) will develop radiation-related solid cancer during their lifetimes—and on average, 4002 will develop baseline cancers during their lifetimes. These risks, computed using the procedure defined above, are mathematical expectations of the number of new radiation-attributed and baseline cases. Their uncertainty ranges (CIs) reflect the uncertainties of the estimates as listed in the “Methods” section. The number of cases observed in future would have additional sources of variability (uncertainty), dependent on, e.g., the size of the population group, or future developments in the secular trends in population statistics (on which the risks are based) which are not accounted for here. Further work is currently being done on methods that reduce the dependence, of radiation related risk assessments, on population statistics and survival curves (Ulanowski et al. 2019), which is particularly useful for risk assessments in highly atypical exposed groups (e.g., astronauts, see Walsh et al. 2019). A further source of uncertainty, not directly accounted for here, is related to the basis for the selection of the risk to dose response models applied here in the calculation of lifetime risks. These risk models, mostly from the LSS with publicly available data, are all recent, but a constraint on this selection comes from the requirement to have the full covariance matrix for the models applied here to do the uncertainty analysis. This means that access to the original data is required so that the covariance matrices (not usually published in papers with risk model parameters) can be obtained when new publications become available. A further consideration of multi-model inference using other published models is, therefore, for this reason, a major undertaking and is a suggestion for future work.

In developing the risk assessment framework applied in the WHO Fukushima Health Risk Assessment Report (WHO 2013), the WHO expert panel considered that risk assessment should be based on a comprehensive assessment of all current evidence from all of radiation epidemiology and not only on the epidemiological evidence available from past nuclear accidents such as Chernobyl. Similarly, the authors consider that the risk assessment framework applied in the software tool described here, should also be based on comprehensive assessment of all current evidence from radiation epidemiology. There are several disadvantages to considering only radiation epidemiological evidence from past nuclear accidents. Many of these studies have risks that are compatible with risks from other types of studies (e.g., Thyroid cancer risks in post-Chernobyl studies and the LSS studies, see, e.g., Figure 4 of Jacob et al. 2014)—so other types of studies can provide added weight of evidence to risk-levels determined from post nuclear accident studies. Generally, the ecological study designs that can be applied after nuclear accidents, are not as reliable as other cohort-type study designs applied in other types (e.g., occupational) of studies. Also there have been several meta-analytical studies looking into the effects of low-dose and low dose rates on cancer risks from a broad range of epidemiological studies (e.g., Shore et al. 2017), evidence from studies such as these, would be ignored, if only studies from past-nuclear accidents were considered.

In order to simplify and illustrate this general consideration of applying risk bases for decision making, it is assumed here that either an acute or annual (first-year) effective dose just comes from external exposures in a situation comparable to the Fukushima release. Although exposures to ^131^I occurred in this release, the total thyroid dose ranges were much lower and much narrower than the ranges in either the LSS or after Chernobyl. Furthermore considering the risk contribution from internal and external thyroid exposures, the thyroid cancer incidence ERR after external exposure during childhood to a thyroid dose of 1 Sv in the LSS is very similar to study results of populations exposed to ^131^I after the Chernobyl accident (e.g., see the Fig. 4, upper panel in Jacob et al. 2014). In real life emergency situations the relevant exposure pathways should include internal exposure via inhalation and ingestion pathways. The total dose delivered during the acute period can be dominated by various sources depending on weather conditions, release and fallout properties and isotopic composition, and on countermeasures, such as evacuation or distribution of stable iodine. Total doses accumulated during longer time periods are more likely to have larger contributions from external exposure. Unfolding the effective dose coefficients back to organ doses (as shown in Table 1) is easier to apply for external exposure; therefore, external exposures are considered here for illustrative purposes. Organ doses for internal exposures cannot be deduced from the effective dose coefficients as straightforwardly as for external exposure, because, by definition, the effective dose coefficient is a committed effective dose per unit intake of the parent radionuclide integrated for 50 years after intake, which takes into account retention of the taken radionuclide and its radioactive progeny in the body. Though, in many practical situations for radionuclides with short effective half-life or residence time, such as radioactive isotopes of iodine or caesium, such unfolding can be still reasonably achieved (see, e.g., procedure described in United Nations Effects of Ionizing Radiation 2014, Attachment C-12 and WHO 2013 pp. 134–135, Tables 20 and 21). For radionuclides with long residence time or isotopes of bone-seeking elements (^90^Sr, actinides) such an unfolding may be regarded as implausible and more direct methods of evaluating the organ doses from exposure to such radionuclides need to be applied.

If decisions have to made in the emergency phase of an accident, and radionuclides such as ^131^I are released, then various parts of the human body are inhomogeneously exposed and organ doses may vary considerably. For such exposure situations, translation of effective dose to organ dose can be achieved only for a specific exposure scenario by considering exposure pathways, intake and inhalation, age, sex, diet, location and occupation factors for specific population groups or individuals. However, the aim here was to illustrate how risk bases can be applied to complement decision making based on dose bases, not necessarily to reproduce fully realistic post-accident dosimetric situations which will depend heavily on the type of accident and local conditions at the time of the accident and immediately following. Further work is required to refine the calculations of organ doses from internal exposures that can cause problems in the early stages of a nuclear accident.

In deciding on the types of uncertainties to apply to the dosimetric reference levels, it should be considered that a reference level is an operational intervention level, above which, an action is taken. In that sense, reference levels are deterministic values, with no uncertainties per definition. The uncertainty in the decision making is introduced by comparing assessed or measured doses (that do have uncertainty) with the reference level (without uncertainty). Consequently, an uncertainty has been assigning here to the reference levels in order to consider the uncertainty in the actually assessed doses. The question then is, whether these assessed doses are log-normally distributed or normally distributed. On the one hand, measured doses for an individual (e.g., thyroid absorbed dose) could be expected to follow a normally distributed error, but doses calculated from simulations and estimated source term or assessed from monitoring data could be better represented by log-normally distributed uncertainties. For the purpose of the main results given here, a log-normal distribution was applied with GSD = 1.5 (see, e.g.. Harada et al. 2014) but all tables were also calculated for a normal distribution with a SD which is 20% of the dose reference level (these results tables are not shown). The differences between the risk factors when calculated for both types of dose uncertainties were found to be quite small. As examples of this if one considers the all solid cancer number of lifetime cancers per 10,000 persons, from exposure to 100 mSv at 1 year (i.e., as given in Fig. 5), for males and females the values are 264 (88; 1097) and 416 (164; 1200), respectively; these are 279 (119; 898) and 455 (224; 975) respectively, when calculated assuming that dosimetric errors follow a normal distribution with a SD which is 20% of the dose reference level. In the calculations presented here, the dosimetric reference levels have been assumed to be at the centre of the dosimetric uncertainty treatment. In practice, the reference levels could pertain to maximum doses. In this situation, and based on real life dosimetric data, the CONFIDENCE tool could then be applied treating the dosimetric uncertainties with a realistic dose distribution with the reference levels taken to be the upper 90% or 95% confidence level of the dose distribution. Such an application of the software tool will be considered for further work.

Although current radiological emergency response recommendations have been provided in safety standards and requirements published by the IAEA (2015) and based on the 2007 Recommendations of ICRP (2007), not all countries will adopt the recommendations exactly. Currently accepted dosimetric reference levels vary in different European countries. For example, the UK uses reference levels that are higher than recommended by IAEA of 30–300 mSv whole body dose for evacuation (Ashley et al. 2017). In Germany, the national Commission on Radiological Protection (SSK 2014) has decided to adopt the 2007 ICRP recommendations, and the IAEA safety requirements. Similarly, in Switzerland, the ICRP recommendations and the IAEA safety documents have been adopted (Swiss RPO, 2017—Art 123).

Such differences in national currently accepted dosimetric reference levels can broadly be translated linearly into risk differences, for the same dose metric, for the all solid cancer, breast cancer and thyroid cancer risks presented in this paper– because the LAR estimates are calculated from linear ERR and EAR models. However, the leukaemia LAR estimates would, strictly speaking, need to be recalculated for other reference doses due to the parabolic shape of the leukaemia cancer ERR and EAR dose response applied here (Hsu et al. 2013), but at low doses, the linear component of risk dominates and can, therefore, provide a good approximation of the risk. Based on current epidemiological data, the assumption of linearity in the risk to dose response for solid cancers (i.e., Linear Non-Threshold, LNT), appears to be the most practical and prudent choice for radiation protection purposes (NCRP 2018, Shore et al. 2018). Although the Grant et al. (2017) results for all solid cancer incidence in the LSS indicated that a linear-quadratic ERR dose response model fitted the male data better than the linear model, the linear models were applied in this work for consistency with current radiation protection guidelines based on LNT and the precautionary principle.

The results given in Tables 2, 3, 4 and 5 (and the Figs. 1, 2, 3, 4, 5) show how reference dose levels can translate differently into risks depending on age at exposure, gender, the length of the at-risk time-frame considered and cancer risk type. These results illustrate the potential for such risk-based information to be used by decision makers, in the urgent and transition phases of nuclear emergencies, to identify protective measures (e.g., sheltering, evacuation) in a differential way (i.e., for particularly susceptible sub-groups of a population). For example, sensitive sub-groups of the population can be identified, such as children, for priority consideration. Application of nominal risks provided by ICRP 103 (2007) could in theory also be applied for this purpose, but due to the method of calculation, which involves averaging lifetime risks calculated in 5-year intervals of age at exposure, over age at exposure and averaging over sex, differential risk information is lost. Also, the new software tool presented here may be applied with directly relevant population data for the geographical area at risk. Another advantage of applying risk-bases similar to those presented here is that they include realistic 95% confidence intervals, allowing decision makers to consider best case and worst-case scenarios, before implementing protective measures.

## Conclusions

Due to long delays, in the past, between the occurrence of nuclear accident and the publications of relevant radiation-related health risk assessments, it is useful to have a software tool ready and available, before future accidents occur. The EU-CONFIDENCE tool, described here, is a software tool that can provide risk-based assessments (with uncertainties) potentially important for estimating health effects from external exposures and deciding on interventions such as medical screening actions. Effective medical screening has to be initiated early after the emergency and so risk-based results are recommended for consideration and to be complementary to monitoring and prognosis results. Such a joint consideration of risk bases and dose bases should improve the overall evidence bases on which important radiation protection decisions will need to be made after a nuclear accident.

## Appendix

Supplementary model fitting results are given here for the LSS all solid cancer incidence Excess Absolute Risk (EAR) risk model applied in the software tool. This was necessary because the original publication (for follow-up 1958–2009, Grant et al. 2017) did not provide an EAR model without smoking adjustment [i.e., an EAR model with the same adjustments as the ERR model given as the first entry in Table 5 of Grant et al. (2017)]. Such a model is analogous to the earlier EAR all solid cancer incidence model (for follow-up 1958–1998, Preston et al. 2007) that was found to be very appropriate for and used in the WHO Fukushima risk assessment (WHO 2013). The fit parameters (see Table 6) and parameter covariance matrix for this EAR model, unadjusted for smoking, were obtained using the publicly available data set (rerf.or.jp) and the EPICURE software with the AMFIT module (Preston et al. 1993) for Poisson regression on grouped data.

**Table 6:** Fit parameters for the LSS EAR model considered with the general form $\lambda \left( {d,e,a,s} \right) = \lambda_{0} \left( {e,a,s} \right) + {\text{EAR}}\left( {d,e,a,s} \right)$, where $\lambda$ is the total incidence rate, $\lambda_{0}$ is the baseline incidence rate, *e* is age at exposure, *a* is attained age (both in years), *s* is sex, *d* is the dose (Gy) delivered to the organ/tissue, i.e., colon dose, at age *e*

*All solid cancer* This model was fitted by the current authors using the dataset sol_col_2017ext_v1.csv from http://www.rerf.or.jp, as recently applied (Grant et al. 2017)	${\text{EAR}}\left( {d,a,e,s} \right) = \left( {1 + t s} \right)k_{d} d\exp\left( { - \gamma_{e} \left( {e - 30} \right) + \gamma_{a} \ln \frac{a}{70}} \right)$
	Fit parameters with standard errors are: *t* = 0.1385 ± 0.06223, *k*_*d*_ = 53.31 ± 4.772, $\gamma_{e}$ = 0.03195 ± 0.005086, $\gamma_{a}$ = 2.350 ± 0.2097 (deviance = 57,405.1, *df* = 185,095)
